# A novel DNA damage repair gene-related prognostic model for evaluating the prognosis and tumor microenvironment infiltration of esophageal squamous cell carcinoma

**DOI:** 10.1186/s12920-023-01459-1

**Published:** 2023-02-20

**Authors:** Dong Guo, Xueyuan Zhang, Xingyu Du, Weinan Yao, Wenbin Shen, Shuchai Zhu

**Affiliations:** Department of Radiation Oncology, Fourth Hospital of Hebei Medical University, Shijiazhuang, 050000 China

**Keywords:** Esophageal squamous cell carcinoma, DNA damage repair, Risk score, Prognosis, Immune

## Abstract

**Background:**

This study aimed to investigate the potential prognostic value of DNA damage repair genes (DDRGs) in esophageal squamous cell carcinoma (ESCC) and their relationship with immune-related characteristics.

**Methods:**

We analyzed DDRGs of the Gene Expression Omnibus database (GSE53625). Subsequently, the GSE53625 cohort was used to construct a prognostic model based on least absolute shrinkage and selection operator regression, and Cox regression analysis was used to construct a nomogram. The immunological analysis algorithms explored the differences between the potential mechanism, tumor immune activity, and immunosuppressive genes in the high- and low-risk groups. Of the prognosis model-related DDRGs, we selected PPP2R2A for further investigation. Functional experiments were conducted to evaluate the effect on ESCC cells in vitro.

**Results:**

A 5-DDRG (ERCC5, POLK, PPP2R2A, TNP1 and ZNF350) prediction signature was established for ESCC, stratifying patients into two risk groups. Multivariate Cox regression analysis showed that the 5-DDRG signature was an independent predictor of overall survival. Immune cells such as CD4 T cells and monocytes displayed lower infiltration levels in the high-risk group. Additionally, the immune, ESTIMATE, and stromal scores in the high-risk group were all considerably higher than those in the low-risk group. Functionally, knockdown of PPP2R2A significantly suppressed cell proliferation, migration and invasion in two ESCC cell lines (ECA109 and TE1).

**Conclusion:**

The clustered subtypes and prognostic model of DDRGs could effectively predict the prognosis and immune activity of ESCC patients.

**Supplementary Information:**

The online version contains supplementary material available at 10.1186/s12920-023-01459-1.

## Introduction

Esophageal cancer is one of the most common malignant tumors and seriously threatens human health and well-being [1]. In Asia, esophageal squamous cell carcinoma (ESCC) is the main histologic type of esophageal cancer [2]. Although we have made great progress in multimodality therapies for ESCC, such as surgery, chemoradiotherapy, and targeted drug therapy, the unsatisfactory clinical outcomes of ESCC have not improved appreciably [3]. Immunotherapy has a promising future, and progress has been made in the combined management of ESCC patients [4]. However, resistance to immunotherapy may elicit low response rates with subsequent tumor recurrence and metastasis. Hence, exploration of the underlying biological mechanisms of tumorigenesis and identification of new targets of immunotherapies to improve the prognosis of ESCC are urgently needed.

Various environmental and endogenous risk factors, such as ionizing radiation (IR), alkylating agents, antimetabolites and other chemical factors, can trigger DNA damage [5, 6]. DNA damage repair (DDR) pathways are a network of cellular signaling pathways that recognize and repair DNA damage. The interaction of these DDR pathways can prevent gene distortion and accumulation of DNA damage and ensure the integrity of the genome [7]. Dysfunction of the DDR process promotes cell aging, apoptosis, and tumorigenesis [8, 9]. Lawrence et al. indicated that the balance between DNA damage and DNA repair ability may facilitate genome distortion in malignant cells. Distinguishing these tumor cells from normal cells can improve the treatment response of cancer [10]. Additionally, DDR gene (DDRG) mutations usually result in a high somatic mutation load in malignant cells, and these variations in turn trigger the production of tumor-specific neoantigens [11, 12]. DDRG research can broaden therapy options by exploring the characteristics of gene drivers for cancer patients in translational medicine. McLaughlin et al. found that DDRG mutations could alter inflammation-related signaling pathways, which could reshape the tumor immune status [13]. More importantly, DDRG mutations are emerging as potential biomarkers for predicting tumor prognosis and immunotherapeutic response. Song et al. revealed that DDRG mutations were indicative of a favorable prognosis in colorectal cancer [14]. Chae et al. found that a DDRG signature was predictive of patient prognosis in glioma cells [11]. In addition, studies have shown that high DDRG mutation loads are closely related to CD4+ and CD8+ tumor-infiltrating lymphocytes (TILs), and the co-mutations of homologous recombination repair and mismatch repair (HRR-MMR) in the DDR pathways are potential biomarkers for immune checkpoint inhibitor (ICI) therapy[15]. Although these findings have highlighted the importance of the DDRG signature for tumor prognosis and immunotherapeutic response, the prognosis and immune features of DDRGs in ESCC remain unclear.

To address this issue, we investigated the DDRG signature of ESCC patients and utilized the Gene Expression Omnibus (GEO) and The Cancer Genome Atlas (TCGA) databases to construct a clustering subtype and risk score model. We first examined the predictive ability of the DDRG signature for ESCC patients. Then, we evaluated the relationship between the DDRG signature and immune-related characteristics. Finally, we conducted cell experiments to further verify the expression of DDRGs (ERCC5, POLK, PPP2R2A, TNP1 and ZNF350). Our results demonstrated that the DDRG signature had significant advantages in predicting prognosis and distinguishing hot tumors to advance the individualization of immunotherapy.

## Materials and methods

### Data acquisition

The expression profiles for GSE53625 and clinical data were obtained based on the GPL18109 platform from the GEO (https://www.ncbi.nlm.nih.gov/geo/) database, which was used as a training cohort. In addition, as an external validation cohort, we extracted RNA-FPKM data and clinical data of TCGA-ESCC from the TCGA database (https://tcga-data.nci.nih.gov/tcga/). ESCC samples with complete clinical outcome time and status were retained in this study. DDRGs were identified from the Molecular Signatures Database (MSigDB, https://www.gseamsigdb.org/gsea/msigdb) and previously published literature[16].

### Establishment and validation of the prognostic model

The prognostic DDRGs with *P* < 0.05 in the GSE53625 cohort were screened in univariate Cox regression analysis through the “survminer” and “survival” R packages [17, 18]. Subsequently, the least absolute shrinkage and selection operator (LASSO) regression algorithm with 10 times cross validation was performed to construct a risk model by using the “glmnet” R package. The risk score was determined by LASSO regression coefficients following the formula: risk score = $\sum _{1}^{i}($gene Expression∗ gene coefficient). ESCC patients were divided into two risk groups (high- and low-risk groups).

We analyzed the association between the risk score and clinical characteristics, such as age, gender, grade, T stage, N stage, M stage, and TNM stage, using the “ComplexHeatmap” R package. Kaplan‒Meier curves of OS were generated between the two risk groups. Receiver operator characteristic (ROC) curve analysis (including 1-, 3-, and 5-year survival) was plotted to estimate the predictive efficacy of the risk model using the “timeROC” R package[19]. External validation was conducted in the TCGA-ESCC cohort to test the stability of the risk score application.

### Independent prognostic analysis and nomogram construction

Univariate and multivariate Cox regression analyses were conducted to analyze the risk score and clinical characteristics of the GSE53625 cohort, and the *P* value, HR and 95% CI of each enrolled variable were displayed using the “forestplot” R package. Subsequently, the “rms” R package was used to generate a nomogram predictive of OS based on independent prognostic criteria[20]. A nomogram showed the intuitive results of the risk score. Calibration curves were drawn to evaluate the predictive accuracy of survival probabilities (including 1-, 3-, and 5-year survival).

### Analysis of biological property and pathway enrichment

Utilizing the “clusterProfiler” R package, Gene Ontology (GO) and Kyoto Encyclopedia of Genes and Genomes (KEGG) [21–23] analyses of significantly differentially expressed DDRGs were carried out to assess the biological functions and signaling mechanisms. Moreover, gene set enrichment analysis (GSEA) was conducted to determine different biological functions among the two risk groups of patients. Gene set items with normalized *P* < 0.05 and FDR < 0.25 were regarded as statistically significant.

### Assessment of the immune microenvironment

The “tidyverse” R package was used to explore the correlation of tumor-infiltrating immune cells and immunosuppressive genes with 5 prognostic model-related DDRGs, and the results are displayed in a heatmap. Comparisons of the StromalScore, ImmuneScore, and ESTIMATEScore among the two risk groups in the training cohort were calculated using the “ESTIMATE” R package.

### Cell culture and transfection

Human esophageal epithelial cells (HEEC) and ESCC cells (ECA109 and TE1) were obtained from the Scientific Research Center of the Fourth Hospital of Hebei Medical University (Shijiazhang, China). Cells were cultured in RPMI 1640 medium (Gibco), supplemented with 10% fetal bovine serum (FBS) and placed in a 37 °C, 5% CO2 incubator. Small interfering (si-PPP2R2A) and negative control (si-NC) siRNAs were used for ESCC cell transfection by RIBOBRO (Guangzhou, China). Following the manufacturer’s instructions, Lipofectamine^®^ 2000 (Invitrogen, USA) was used for transfection. After 48 h, the biological functions of transfected ESCC cells were evaluated.

### Quantitative realtime PCR analysis

Total cell RNA was extracted using TRIzol reagent (Thermo Fisher Scientific), and we used a RevertAid First Strand cDNA Synthesis Kit (Thermo Fisher Scientific) to reverse-transcribe to cDNA. MonAmp™ SYBR^®^ Green qPCR Mix was used to perform qRT-PCR to measure the expression of the survival-related DDRGs in esophageal cells. Glyceraldehyde phosphate dehydrogenase (GAPDH) was analyzed as an internal control. The 2^−ΔΔCT^ method was utilized to analyze survival-related DDRG expression. The primer sequences are listed in Table 1.

**Table 1:** The primer sequences of five DDRGs used for qRT-PCR.

Genes	Forword sequence (5′–3′)	Reverse sequence (5′–3′)
ERCC5	CACCAAGCGCAGAAGAACATT	ACCACTCTCCTTGACTCTACCT
POLK	ACTTTGACAAATACCGAGCTGTG	GGAGAGATGGATCGTTCATGC
PPP2R2A	GCGAGACATAACCCTAGAAGC	CACTTGCACAGACTTTGCGAG
TNP1	GACCAGCCGCAAATTAAAGAGT	GGTTGCCCTTACGGTATTTTCT
ZNF350	GCTTGCAGAGTGAAAGCCTG	TCCCCATTTCCAGTAAACTCAAC

*DDRGs* DNA damage repair genes

### Cell counting kit-8 (CCK-8) proliferation assay

The proliferation ability of ESCC cells was evaluated using the CCK-8 kit (Med Chem Express Princeton, USA). After 48 h of transfection, transfected ECA109 and TE1 cells were inoculated into 96-well plates at a density of 4000 cells per well. At 24, 48, 72, and 96 h, the transfected cells were incubated with 10 µl CCK-8 solution for 2 h. The absorbance in each well for living cells was examined at 450 nm wavelength using a microplate reader.

### Wound-healing assay

Transfected ECA109 and TE1 cells were seeded into a 6-well plate. When these cells reached 80% confluence, they were scratched with sterile 200-µl pipette tips. Subsequently, serum-free medium was added to the plates with living cells for 24 h, and the wound healing distance was analyzed under a microscope and photographed.

### Transwell assay

A total of 2.0 × 10^5^ cells were placed in the upper chamber with Matrigel, and the cells were cultured in serum-free medium. Then, 600 µl of medium with 20% FBS was added to the lower chamber. After 24 h, these cells migrated across the Matrigel in the upper chamber and were fixed with paraformaldehyde and stained with crystal violet for further analysis. The stained cells on the lower surface of the filter were counted and imaged using a light microscope.

### Statistical analysis

Student’s *t test* was employed to assess the difference between two groups with regard to ImmuneScore, StromalScore, and ESTIMATEScore. We examined the correlation between two variables using Spearman’s correlation analysis. Survival differences were determined using the Kaplan‒Meier method and compared by log-rank tests. R software (version 4.1.2) was used for all bioinformatics analyses and statistical analyses and to generate the corresponding figures. *P* less than 0.05 denoted statistically significant differences.

## Results

### Identification of prognostic DDRGs

**Fig. 1:**
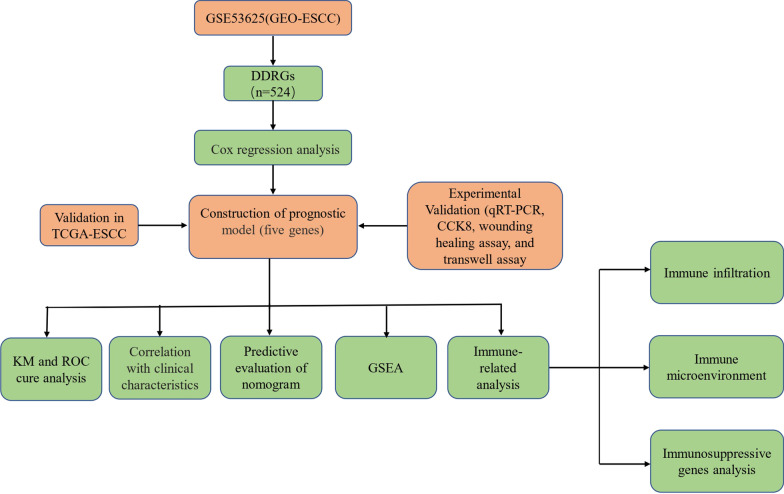
The flow chart of research process

**Fig. 2:**
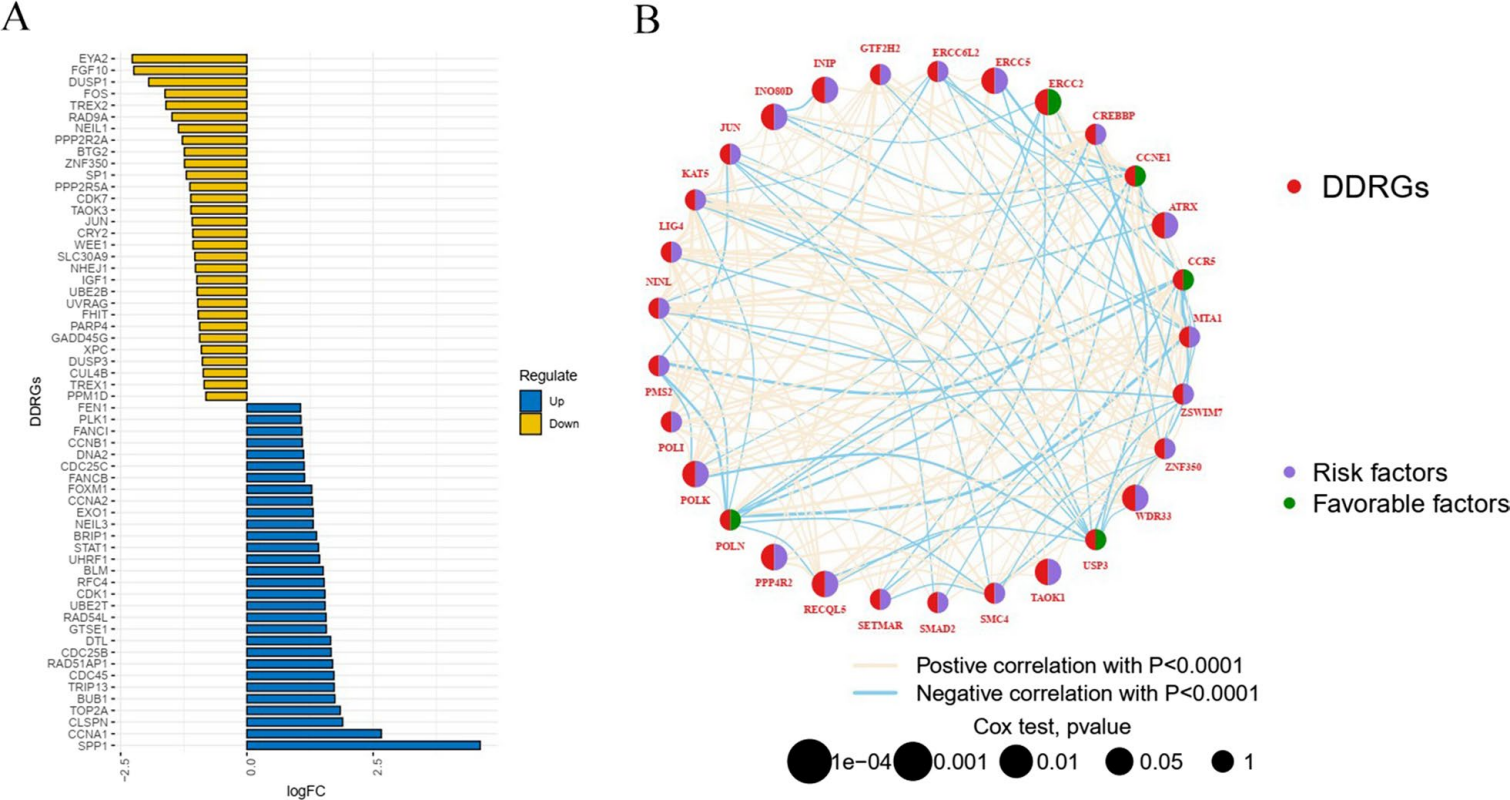
The genetic landscape of DDRGs of ESCC in GSE53635 cohort. **A** The of differentially expressed DDRGs between ESCC and normal samples. **B** The gene interaction network of DDRGs in ESCC. The circle represents its role (purple circle: risk factor; green circle: favorable factor). red line: positive association; blue line: negative relationship). The pink lines or blue lines between genes means they had positive association or negative association. DDRGs, DNA damage repair genes; ESCC, esophageal squamous cell carcinoma

The flowchart summarizes the procedures of the present work (Fig. 1). Compared to normal samples, abnormal expression levels of DDRGs were observed in ESCC samples (Table 2). The analysis included the GSE53625 cohort and TCGA-ESCC cohort. We analyzed differentially expressed DDRGs in the GSE53625 cohort (179 ESCC vs. 179 normal samples) and used the Wilcoxon test for subsequent analysis. In total, 135 differentially expressed DDRGs were identified for investigation (|logFC|>1, FDR < 0.05). Seventy DDRGs were downregulated, and 65 were upregulated. The top 30 downregulated and upregulated DDRGs are visualized in Fig. 2A. In addition, prognostic analysis was performed for DDRGs in the GSE53625 cohort. The network diagram of prognostic DDRGs indicated that ERCC2, CCNE1, CCR5, USP3, and POLN were favorable factors for OS, and others were risk factors for OS (Fig. 2B).

**Table 2:** The differentially expressed DDRGs in normal and ESCC samples

Gene	Normal	ESCC	*P* value	Gene	Normal	ESCC	*P* value
GTSE1	9.744	11.317	3.33E−56	MEIOB	10.394	9.790	6.92E−09
AGGF1	11.743	11.061	1.72E−30	MMS22L	9.297	10.074	3.65E−31
DUSP1	15.033	13.088	1.74E−54	NABP1	11.340	10.732	5.54E−09
CCR5	7.964	7.200	3.64E−13	NEIL1	10.445	9.089	1.20E−41
ATMIN	13.220	12.427	1.49E−50	NEIL3	6.700	8.013	1.77E−23
ATXN3	9.974	9.267	2.95E−18	NHEJ1	8.895	7.876	1.35E−25
BARD1	11.984	11.378	8.04E−28	NINL	11.110	10.338	2.13E−20
BLM	8.873	10.383	6.28E−46	NUDT1	12.530	13.440	1.04E−30
BRCA1	10.519	11.457	1.94E−36	PARP4	9.751	8.813	1.53E−10
BRCA2	8.670	9.378	1.21E−21	PCNA	12.672	13.431	4.58E−34
BRCC3	11.317	10.698	3.73E−38	PLK1	10.960	12.030	2.64E−45
BRIP1	7.656	9.034	1.96E−32	POLD4	14.549	13.786	3.19E−23
BTG2	13.520	12.281	4.27E−38	POLE2	9.487	10.153	1.96E−17
BUB1	9.946	11.689	4.25E−33	POLI	11.400	10.811	2.03E−18
CCNA1	7.522	10.184	3.59E−30	POLK	11.168	10.545	5.03E−31
CCNA2	11.757	13.054	6.88E−39	POLN	7.020	6.336	4.42E−08
CCNB1	11.660	12.758	1.67E−40	PPM1D	11.082	10.271	7.42E−30
CDC25A	8.295	9.064	1.73E−15	PPP2R2A	13.297	12.018	3.50E−44
CDC25B	10.210	11.877	4.46E−50	PPP2R5A	13.087	11.958	1.06E−25
CDC25C	8.491	9.624	1.68E−44	PRKDC	12.513	13.227	1.99E−34
CDC45	10.375	12.099	2.09E−47	PTTG1	12.931	13.548	4.36E−15
CDH13	9.468	10.192	3.88E−11	RAD51AP1	9.171	10.869	3.92E−53
CDK1	10.831	12.377	1.93E−48	RAD54B	9.113	10.016	2.87E−36
CDK4	12.366	13.145	2.44E−37	RAD54L	10.165	11.733	4.01E−41
CDK7	14.089	12.978	5.99E−35	RAD9A	11.588	10.104	1.64E−51
CHAF1A	10.342	10.965	9.45E−30	RECQL4	12.383	13.145	2.21E−14
CHAF1B	10.370	11.010	9.84E−27	REV1	11.355	10.612	2.53E−27
CHEK1	11.931	12.679	3.88E−29	RFC3	10.144	11.015	1.78E−24
CIB1	13.365	12.627	3.61E−26	RFC4	11.997	13.526	1.03E−48
CLSPN	7.201	9.096	5.75E−45	RFC5	11.447	12.055	1.60E−24
CRY1	10.617	9.813	2.86E−14	RNASEH2A	11.945	12.998	4.35E−42
CRY2	12.137	11.066	1.59E−46	RNF168	10.374	11.067	5.69E−22
CUL4B	13.242	12.377	8.60E−48	RNF8	8.233	7.593	6.33E−23
CYP1A1	6.559	5.822	7.60E−09	RPA3	12.129	12.799	8.80E−26
DBF4	4.439	5.490	1.34E−21	RUVBL1	11.495	12.553	4.21E−46
DCLRE1C	10.418	9.776	6.82E−25	SETD2	12.373	11.570	1.70E−54
DNA2	8.228	9.352	3.33E−42	SETMAR	11.561	10.973	4.23E−25
DTL	10.019	11.677	1.22E−54	SIRT1	11.850	11.156	1.08E−29
DUSP3	13.259	12.376	3.89E−19	SLC30A9	11.373	10.342	3.20E−43
E2F1	10.531	11.118	1.84E−19	SMARCA2	13.332	12.564	2.34E−24
ERBB2	11.075	10.489	4.21E−21	SP1	13.510	12.309	3.77E−51
ERCC5	10.151	9.497	9.02E−25	SPP1	9.610	14.230	1.28E−56
ESCO2	9.036	10.011	3.25E−26	STAT1	12.839	14.257	5.76E−41
ESR1	8.717	8.121	6.35E−16	TAOK3	12.418	11.310	2.71E−55
EXO1	10.827	12.138	1.17E−52	TERF2IP	9.114	8.355	5.29E−33
EYA2	13.142	10.872	3.91E−44	TICRR	8.583	9.248	1.31E−19
EYA3	10.520	9.724	2.67E−35	TNP1	10.960	10.292	5.48E−15
EYA4	5.182	6.047	2.56E−10	TONSL	9.696	10.340	8.57E−21
FANCA	7.853	8.592	1.56E−31	TOP2A	11.878	13.727	1.62E−58
FANCB	8.769	9.910	2.64E−34	TOPBP1	11.568	12.325	1.66E−34
FANCI	11.166	12.256	3.52E−52	TREX1	12.628	11.781	1.97E−18
FEN1	10.115	11.178	9.32E−33	TREX2	9.446	7.841	1.05E−23
FGF10	8.873	6.632	1.73E−39	TRIP13	10.141	11.869	5.47E−56
FHIT	11.480	10.515	2.28E−28	UBE2B	11.795	10.809	8.27E−36
FIGN	7.195	6.422	1.96E−14	UBE2D3	12.953	12.254	1.47E−46
FOS	15.140	13.518	1.65E−33	UBE2T	10.341	11.889	8.45E−44
FOXM1	9.599	10.882	9.85E−49	UHRF1	10.224	11.663	4.26E−53
GADD45G	11.536	10.600	2.68E−19	UIMC1	11.957	11.317	1.83E−30
GEN1	9.948	10.635	1.66E−17	USP47	11.594	10.831	2.65E−26
H2AFX	10.946	11.533	8.22E−10	USP7	12.000	11.329	2.50E−40
HELQ	10.542	9.816	1.35E−39	UVRAG	9.776	8.802	1.40E−23
IFI16	13.902	14.791	3.24E−39	UVSSA	10.234	9.590	2.03E−30
IGF1	12.324	11.333	3.35E−09	WDR48	11.546	10.773	8.86E−39
INO80D	12.490	11.742	8.17E−39	WEE1	12.113	11.047	5.21E−34
IRS1	9.332	10.156	6.73E−17	WWP1	13.051	12.464	2.33E−34
JUN	13.709	12.626	1.78E−32	XPC	12.710	11.807	2.47E−45
KAT5	12.951	12.291	4.73E−24	ZNF350	12.084	10.848	5.02E−33
KPNA2	13.080	10.516	1.39E−51				

*DDRGs* DNA damage repair genes, *ESCC* Esophageal squamous cell carcinoma

### Identification of the DDR-related prognosis model

Based on the GSE53625 cohort, univariate Cox regression was performed to determine DDRGs affecting survival. A total of 5 prognostic DDRGs (*P* < 0.05, |HR|>1) were included in the LASSO Cox regression model analysis, including ERCC5, POLK, PPP2R2A, TNP1 and ZNF350 (Additional file 1: Figure S1A–B). A risk model was established based on the expression of 5 DDRGs and their corresponding coefficients: (0.31853) * ERCC5 + (0.28215) * POLK + (− 0.3054) * PPP2R2A + (0.1808) * TNP1 + (0.2069) *  ZNF350 (Additional file 1: Figure S1C). Each patient was divided into a high- or low-risk group according to the optimum cutoff value obtained by means of the “survminer” R package. Fifty genes and their 5 co-expressed DDRGs are depicted in a Sankey plot. The results showed that there was a positive regulatory relationship between 50 genes and their co-expression DDRGs (Additional file 1: Figure S1D).

### Correlation analysis of the prognostic model with clinical outcomes

**Fig. 3:**
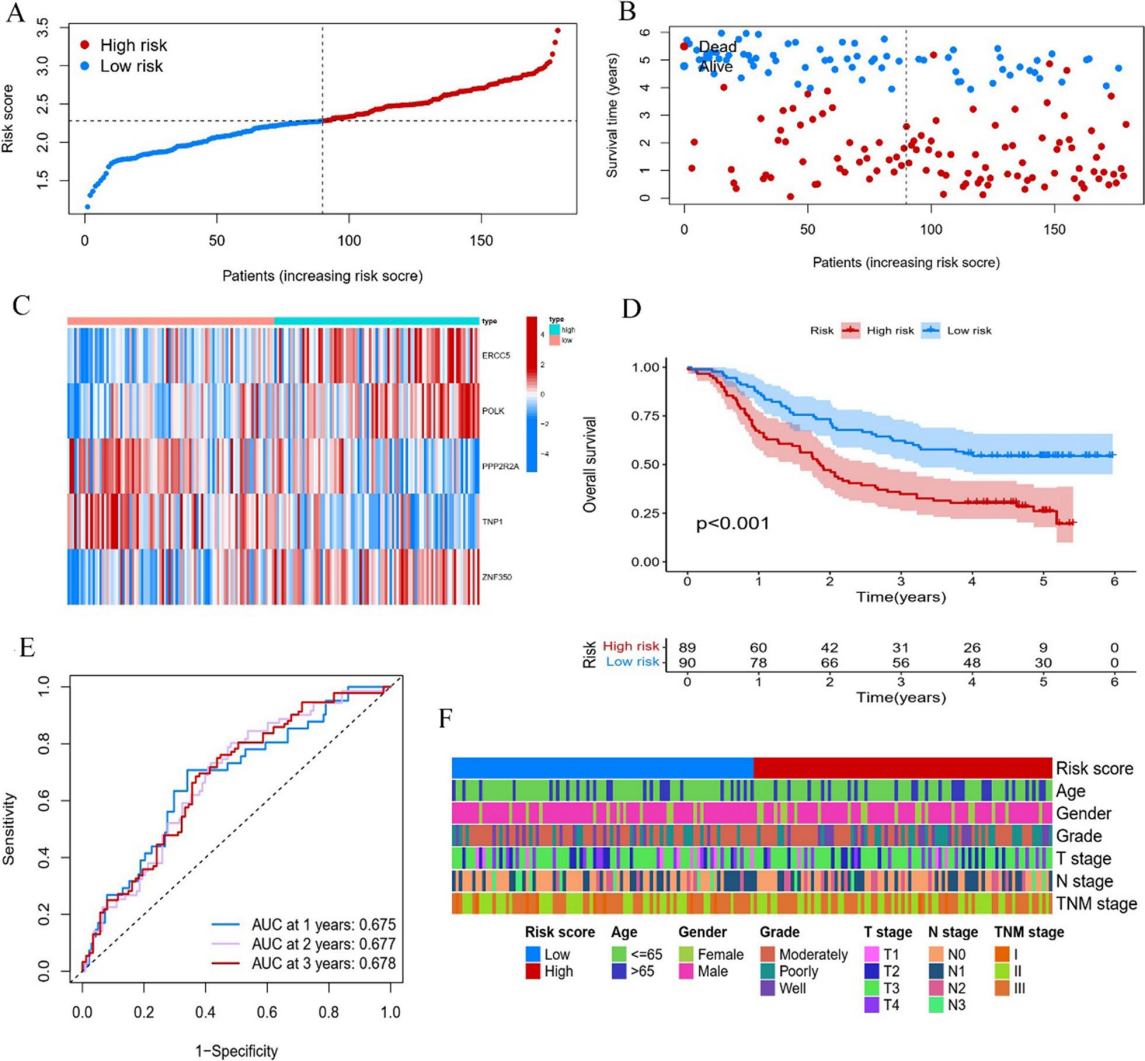
Generation of a DDR-related prognostic model for ESCC in the training cohort. **A**, **B** Distribution of risk scores and survival status of ESCC patients. **C** Heat map showing the association of risk score and 5 DDRGs of ESCC patients. **D** Kaplan-Meier analysis of OS in the high and low-risk groups. **E** ROC curves of DDR-related prognostic model illustrating the prediction efficiency for 1-, 2-, and 3-year survival. **F** Distribution of clinicopathological features in the two risk groups. *DDR* DNA damage repair, *ESCC* esophageal squamous cell carcinoma, *DDRGs* DNA damage repair genes, *OS* Overall survival, *ROC* Receiver operator characteristic

As shown in Fig. 3A–B, patients with a higher percentage of death events were observed in the high-risk group. The heatmap showed that ERCC5, POLK, PPP2R2A, TNP1 and ZNF350 expression levels had significant correlations with risk scores (Fig. 3C). The survival rate of the high-risk patients was significantly poorer than that of the low-risk patients (*P* < 0.001, Fig. 3D), which is consistent with previous reports. The area under the curve (AUC) values for 1-, 2-, and 3-year prognostic prediction were 0.675, 0.677, and 0.678, respectively (Fig. 3E). There was a significant survival difference between patients among the two risk groups (*P* < 0.001, Fig. 3F). In addition, the results showed that the correlation between the risk score and other clinical characteristics was not statistically significant (all *P* > 0.05, Table 3).

**Table 3:** Clinicopathological characteristics of the GSE53625 cohort in prognostic model

Variables	Prognostic model		*P* value
	High risk group	Low risk group	
*Age*			0.583
≤ 65	63 (70.8)	67 (74.4)	
> 65	26 (29.2)	23 (25.6)	
*Gender*			0.166
Male	69 (77.5)	77 (85.6)	
Female	26 (29.2)	13 (14.4)	
*Grade*			0.223
Well/Moderate	61 (68.5)	69 (76.7)	
Poor	28 (21.5)	21 (23.3)	
*T stage*			0.387
T1–T2	17 (19.1)	22 (24.4)	
T3–T4	72 (70.9)	68 (85.6)	
*N stage*			0.676
N0–N1	71 (79.8)	74 (82.0)	
N2–N3	18 (20.2)	16 (18.0)	
*TNM stage*			0.707
I–II	42 (47.2)	45 (50.0)	
III	47 (52.8)	45 (50.0)	
*Status*			< 0.001
Dead	65 (73.0)	41(45.6)	
Alive	24 (27.0)	49 (54.4)	

### Verification of the prognostic model

**Fig. 4:**
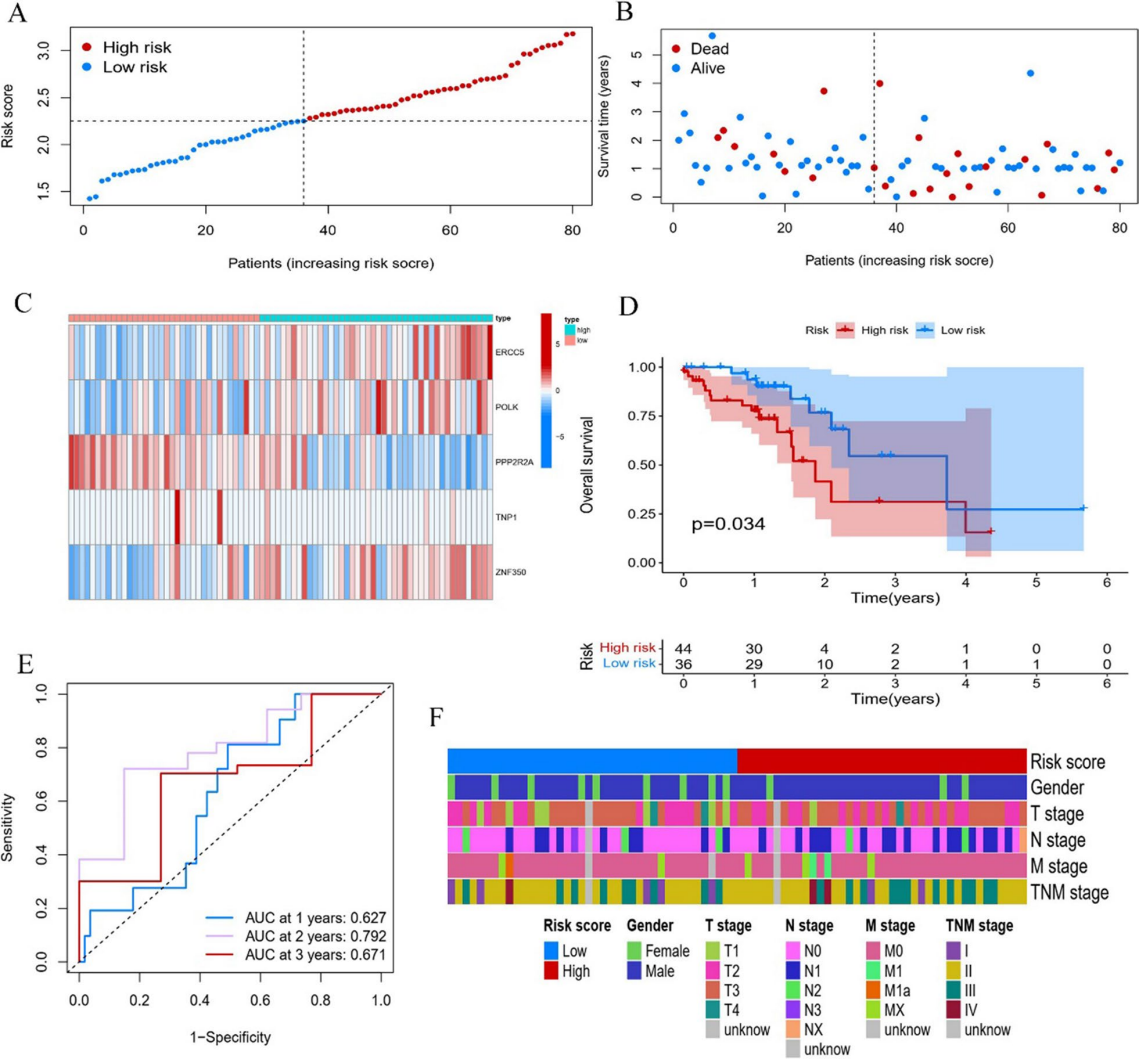
Generation of a DDR-related prognostic model for ESCC in the validation cohort. **A**, **B** Distribution of risk scores and survival status of ESCC patients. **C** Heat map showing the association of risk score and 5 DDRGs of ESCC patients. **D** Kaplan‒Meier analysis of OS in the high and low-risk groups. **E** ROC curves of DDR-related prognostic model illustrating the prediction efficiency for 1-, 2-, and 3-year survival. **F** Distribution of clinicopathological features in the two risk groups. *DDR* DNA damage repair, *ESCC* Esophageal squamous cell carcinoma, *DDRGs* DNA damage repair genes, *OS* Overall survival, *ROC* Receiver operator characteristic

The TCGA-ESCC cohort was analyzed as an external validation cohort to confirm the predictive values of the developed prognostic model. Based on the median risk score in the GSE53625 cohort, 80 patients in the TCGA-ESCC cohort were divided into the high-risk group (44 patients) and low-risk group (36 patients). The distribution characteristics of ESCC patients in the TCGA-ESCC cohort are shown in Fig. 4A–B. A heatmap showed that the expression level varied systematically among prognostic model-related DDRGs (Fig. 4C). Kaplan‒Meier survival curves demonstrated that the prognostic model could remarkably distinguish the clinical outcomes (*P* = 0.034, Fig. 4D). In the TCGA-ESCC cohort, ROC curve analysis showed that the AUCs of the 1-, 2-, and 3-year OS were 0.627, 0.792, and 0.671, respectively (Fig. 4E). In addition, the heatmap displayed the relationship between the risk score and clinical characteristics (Fig. 4F).

### Construction of a prognostic nomogram

**Fig. 5:**
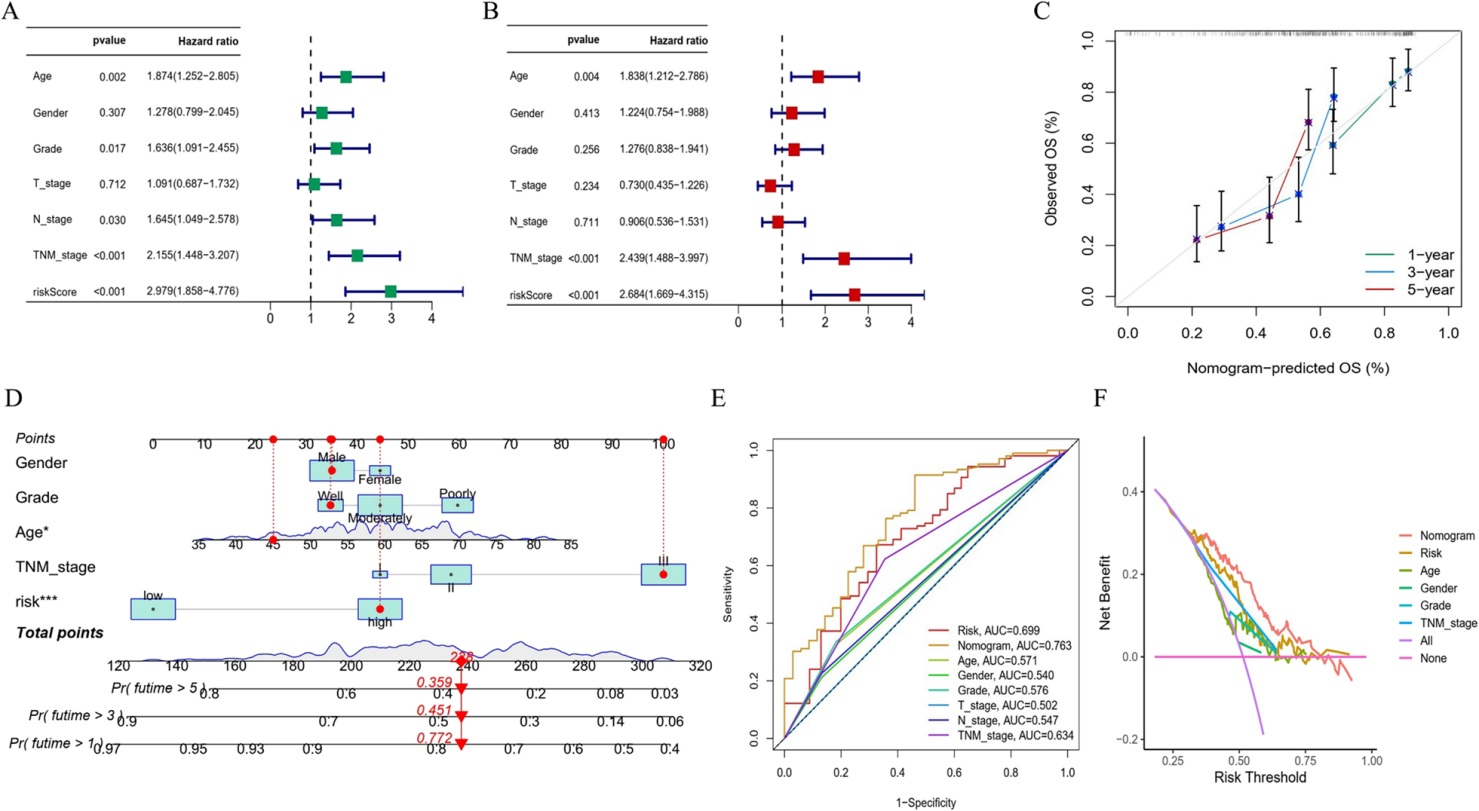
Construction of a nomogram predicting the OS of ESCC patients. **A**, **B** Univariate and multivariate Cox regression analysis for the prognostic signature. **C** The calibration plots of the nomogram for predicting the 1-, 3-, and 5-year survival of ESCC patients. **D** Construction of a nomogram with risk score and clinicopathological features. **E** The ROC curves of the nomogram and clinicopathological features. **F** Decision curve analysis. *OS* overall survival, *ESCC* Esophageal squamous cell carcinoma, *ROC* Receiver operator characteristic

To evaluate whether the 5-DDRG prognostic model could be an independent predictor affecting ESCC clinical outcomes, we established a prognostic nomogram combining risk scores with clinical characteristics. The univariate and multivariate Cox regression analyses of the GSE53625 cohort indicated that patient age (*P* = 0.004, HR = 1.838), TNM stage (*P* < 0.001, HR = 2.439) and the constructed risk score (*P* < 0.001, HR = 2.684) were independent prognostic indicators in ESCC patients (Fig. 5A‒B). Based on the results above, a nomogram was established to predict survival at 1, 3 and 5 years (Fig. 5C). A prognostic nomogram was constructed based on the proportion of contribution to the death risk, as shown in Fig. 5D. The AUC of the nomogram for predicting OS was 0.763, which was higher than the values of the risk score and other clinical characteristics (Fig. 5E). Decision curve analysis (DCA) confirmed that this model had the highest net benefit, suggesting that this model can be effectively applied for clinical decision-making (Fig. 5F).

### Gene set enrichment analyses of the prognostic model

GO and KEGG functional enrichment analyses of the differentially expressed DDRGs among the two risk groups in the GSE53625 cohort were performed to investigate the underlying molecular heterogeneity. For GO analysis, immune-related biological processes (B-cell receptor, positive regulation of DNA binding, humoral immune response mediated by circulating immunoglobulin, T-cell cytokine production and immune response regulating cell surface receptor signaling pathway) were significantly activated in the high-risk group (Additional file 2: Figure S2A). In contrast, the activities of some immune depletion-related pathways (such as pathways related to neutrophil-mediated immunity, inflammatory response to antigenic stimulus, and positive regulation of intrinsic apoptotic signaling pathways) were significantly enriched in the low-risk group (Additional file 2: Figure S2B). The KEGG (www.kegg.jp/kegg/kegg1.html) pathway enrichment results revealed that the genes in the high-risk group were significantly enriched in immune reactivity pathways, such as the TGF-β signaling pathway and antigen processing and presentation (Additional file 2: Figure S2C). Pathways related to linoleic glutathione metabolism and ribosomes converged in the low-risk group (Additional file 2: Figure S2D).

### Immune cell infiltration analysis in the prognostic model

**Fig. 6:**
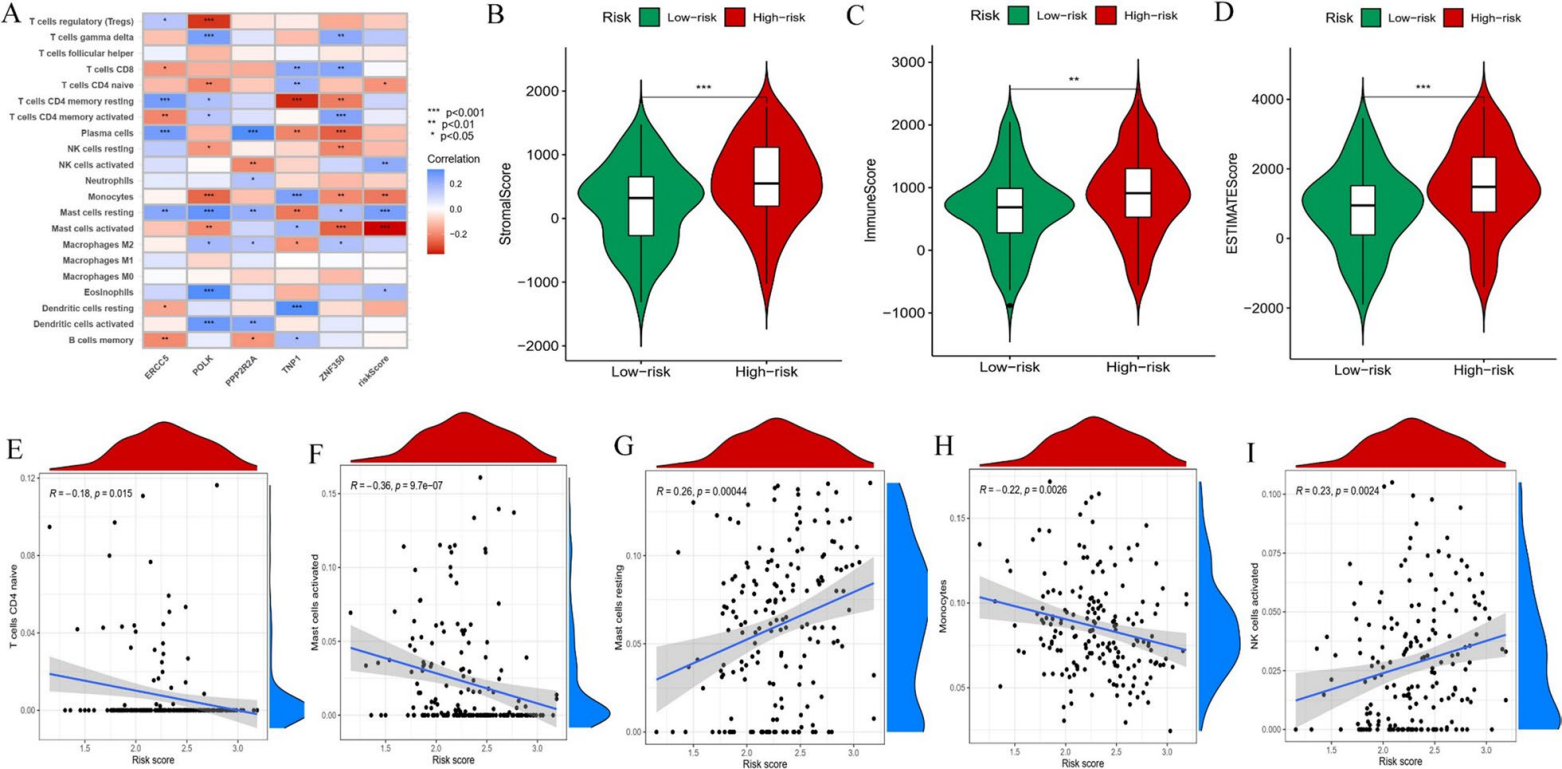
Immunological profiling of different prognostic model. **A** Differences in the distribution of tumor-infiltrating immune cells of different risk groups. **B** The StromalScore, ImmuneScore and ESTIMATEScore in high and low-risk groups. **E‒I** Correlation between the risk score and tumor-infiltrating immune cells. *P < 0.05, **P < 0.01, and ***P < 0.001

We compared the composition of 22 infiltrating immune cells among the two risk groups in the GSE53625 cohort. The results showed that patients in the high-risk group had higher infiltration levels of activated NK cells and resting mast cells than those in the low-risk group, whereas dendritic cell and macrophage infiltration showed no difference (Fig. 6A). The high-risk group showed a higher StromalScore, ImmuneScore and ESTIMATEScore, which revealed that high-risk patients had higher immunogenicity than the low-risk group (Fig. 6B–D). Additionally, the scatter diagrams further verified the correlation of immune cells (activated NK cells, resting mast cells, activated mast cells, naive CD4 T cells and monocytes) and the risk score (Fig. 6E–I).

**Fig. 7:**
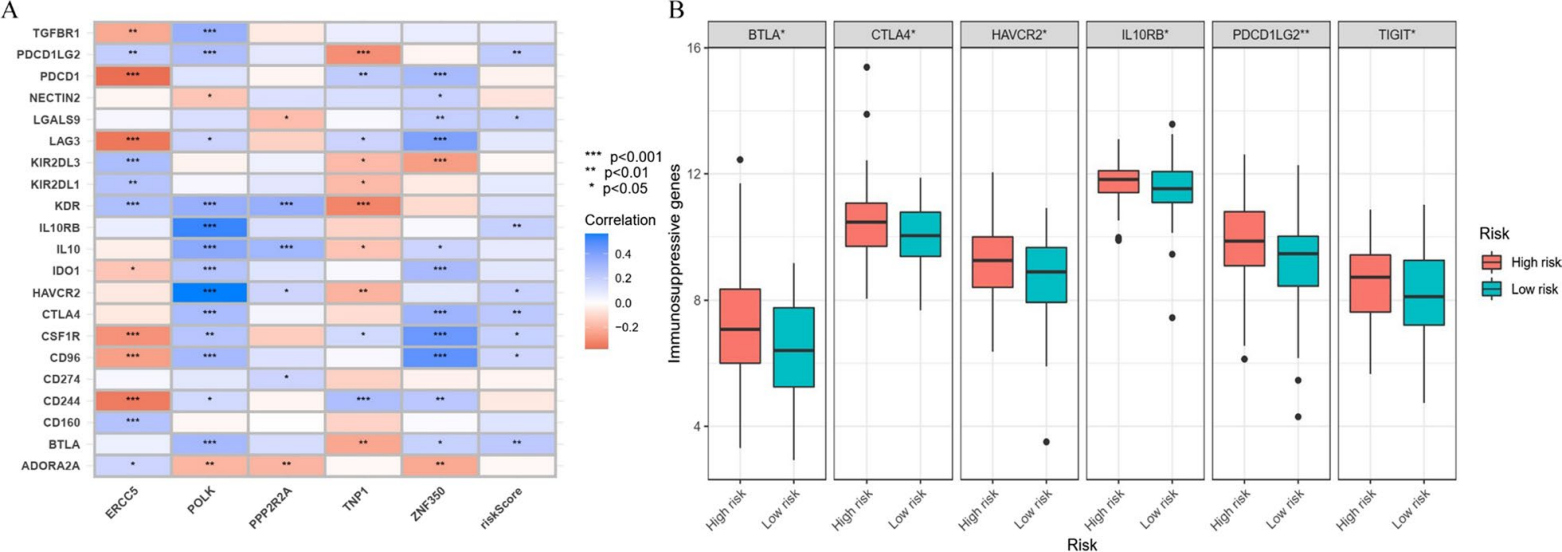
Correlation of the DDR-related prognostic model with the immunosuppressive genes of ESCC patients. **A** Correlation between risk groups and immunosuppressive genes. **B** The expression of BTLA, CTLA4, HAVCR2, IL10RB, PDCD1LG2, and TIGIT in high and low-risk groups. *P < 0.05, **P < 0.01, and ***P < 0.001

We assessed the impact of immunosuppressive genes on ESCC, and the expression of immunosuppressive genes in the two risk groups was analyzed (Fig. 7A‒B). The results indicated that patients in the high-risk group had higher expression of BTLA, CTLA4, HAVCR2, IL10RB, PDCD1LG2, and TIGIT than patients in the low-risk group. These findings revealed that patients with higher risk may benefit from immunotherapies that target these immunosuppressive genes.

### Validation of DDRGs in the prognostic model

**Fig. 8:**
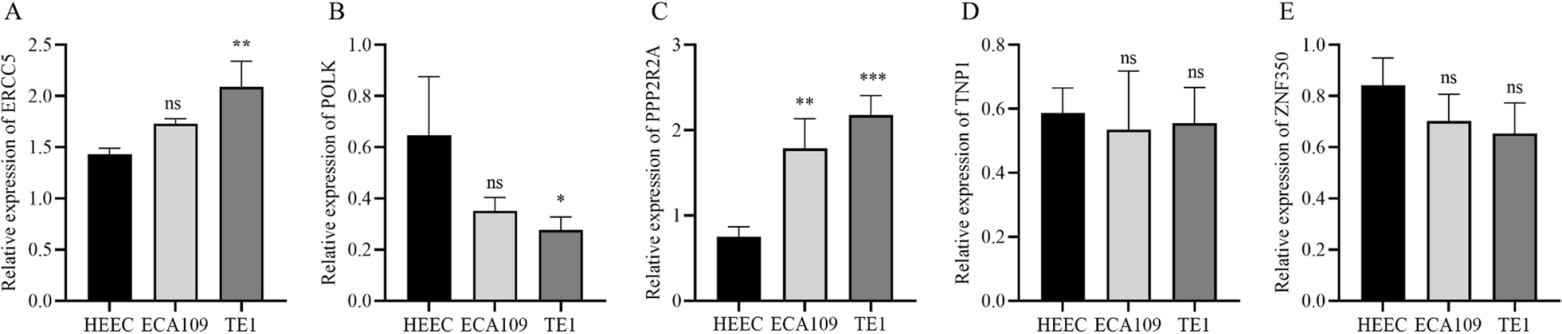
The expression levels of the five DDRGs in cells. **A**–**E** qRT-PCR analysis showing the expression levels of the ERCC5, POLK, PPP2R2A, TNP1 and ZNF350 in the normal esophageal epithelial cells and ESCC cells. *DNA* Damage repair genes, *ESCC* Esophageal squamous cell carcinoma, *qRT-PCR* quantitative real-time polymerase chain reaction, *ns* Not significant; *P < 0.05, **P < 0.01, ***P < 0.001

Quantitative real-time polymerase chain reaction (qRT-PCR) analysis was performed to validate the expression of the prognostic model-related DDRGs in ESCC cells (Fig. 8A‒E). The results indicated that the expression of POLK was downregulated in ESCC cells compared to normal esophageal epithelial cells (HEECs). Additionally, ESCC cells had higher expression levels of ERCC5 and PPP2R2A than HEEC cells.

### The tumorigenic role of PPP2R2A in ESCC in vitro

**Fig. 9:**
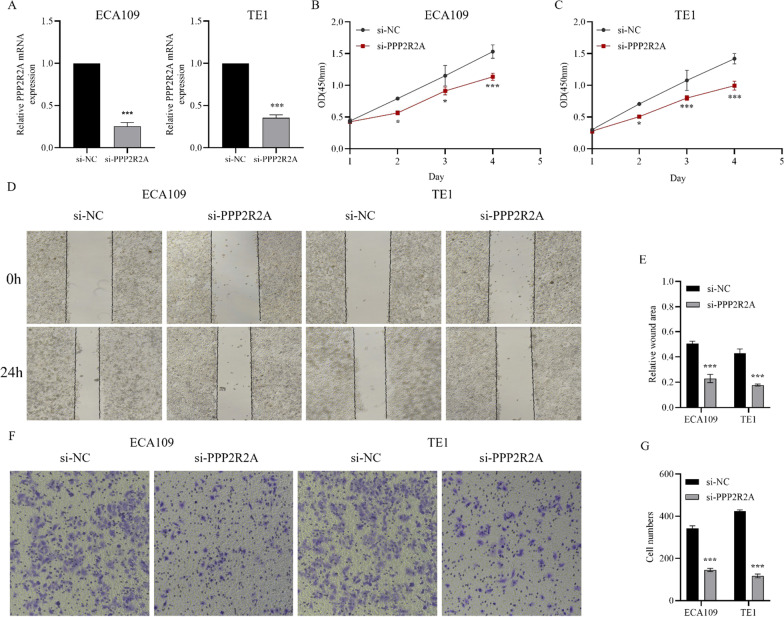
The PPP2R2A’s biological behaviors in ESCC cells. **A** Verification of PPP2R2A knockdown efficiency. **B**, **C** Proliferation curves assessed by CCK8 assay for PPP2R2A knockdown in ESCC cells. **D‒G** Wound healing and transwell assay were performed to confirm the biological function of PPP2R2A in ESCC cells. *ESCC* Esophageal squamous cell carcinoma, *ns* Not significant; *P < 0.05, **P < 0.01, ***P < 0.001

We performed qRT‒PCR to analyze the expression level of PPP2R2A after transfection in ESCC cell lines using targeted siRNA (Fig. 9A). We found that PPP2R2A expression in ECA109 and TE1 cells using si-PPP2R2A was markedly decreased compared with that in the si-NC group. CCK-8 assays revealed that knockdown of PPP2R2A significantly inhibited the proliferation ability of ECA109 and TE1 cells (Fig. 9B, C). In addition, the results of wound healing and transwell assays demonstrated that knockdown of PPP2R2A suppressed ECA109 and TE1 cell migration as well as cell invasion (Fig. 9D‒G). Taken together, our findings suggest that PPP2R2A is involved in the tumorigenesis of ESCC and enhances the proliferation, migration and invasion of ECA109 and TE1 cells in vitro.

## Discussion

ESCC is one of the most common malignant tumors with a high recurrence rate and mortality. Aggressive multimodal therapy has remarkably improved outcomes in patients with ESCC; nevertheless, the treatment outcomes are still unsatisfactory. Primary and acquired therapy resistance remains a major obstacle. A comprehensive analysis of the genetic features is critical for the therapeutic evaluation and prognosis of ESCC. A single gene explaining the molecular signatures of cancer may be farfetched and influenced by confounders. Additionally, some obstacles hamper the prediction accuracy of therapeutic evaluation and prognosis. DDR can maintain the integrity of the genome and homeostasis of cells. In contrast, dysfunction of the DDR process can result in genetic information mutations, eventually leading to malignant transformation. However, the genetic features based on DDRGs in ESCC have not yet been clarified. Herein, we constructed a DDRG molecular signature and comprehensively elucidated its role in the therapeutic evaluation and prognosis of ESCC.

The DDR pathway includes direct repair, mismatch repair, base excision repair, homologous recombination, double-strand break repair, and nonhomologous end-joining, which can accurately repair DDR mutations and maintain genetic stability. DDRGs are often inactivated during the stage of cancer initiation and progression, which suggests that cancer cells exhibit poor DDR capability. Consequently, the accumulation of DNA damage in tumor cells increases dramatically and upregulates mutagenic abnormal proteins[24, 25]. Moreover, these abnormal proteins may act as antigens to drive oncogenesis, which increases the probability of occurrence and development of tumors[26]. It has been reported that different DDRG signatures are closely related to the immune response along with the prognosis of cancer patients. A study in 2021 preliminarily explored the effect of the DDRG PKMYT1 on immunity and prognosis in various malignancies[27]. The results showed that PKMYT1 exerted a vital effect on tumor immunity and progression. Because PKMYT1 acts as an individual gene rather than a DDRG signature, these findings may still have certain limitations. Another study established DDRG signatures in cervical squamous cell carcinoma, and Zhou et al. found that a DDRG signature was associated with prognosis and could act as a biomarker for immunotherapies[28]. Precision immunotherapy based on a DDRG signature should be explored, and its role in the clinical response and prognosis of immunotherapy should be identified.

We obtained tumor sequencing data from publicly available databases (GEO database and TCGA database), which presented the transcriptome profiles of ESCC. In previous studies, these databases have been used to access the genetic landscape and can identify novel biomarkers to predict the prognosis of patients with ESCC[29, 30]. In this study, we screened genes with prognostic significance to construct a prognostic model consisting of 5 genes (ERCC5, POLK, PPP2R2A, TNP1 and ZNF350) in the GSE53625 cohort and validated it in independent TCGA-ESCC cohorts. The patients were classified into high- and low-risk groups based on risk scores, which indicated different individual abilities of DDR. The patients in the high-risk group demonstrated a poor survival time compared to the low-risk group. This prognostic model exhibited high predictive accuracy for ESCC survival, especially for 3-year survival (AUC = 0.678). Although this might result from the limited number of our patients, it is important to further explore the factors for ESCC prognosis. More critically, this risk model showed rationality consistency in TCGA-ESCC cohorts.

The above mentioned 5 genes have attracted extensive attention in various malignancies and were selected for experimental validation in the present study. A recent study demonstrated that ERCC5 (key components of the nucleotide excision repair pathway) was remarkably associated with susceptibility to tumors[31]. Li et al. reported that ERCC5 was significantly associated with the response of cisplatinbased chemotherapy of non-small cell lung cancer[32]. Another study reported that the expression level of ERCC5 was significantly increased in hepatocellular carcinoma, and high ERCC5 expression conferred poor prognosis[33]. POLK is associated with cancer cell proliferation and participates in platinum-chemotherapy tolerance in lung cancer[34]. Considering the higher fold change in the expression level of PPP2R2A, PPP2R2A was selected for further functional assays. Our results revealed that knockdown of PPP2R2A remarkably suppressed cell proliferation, migration and invasion in ECA109 and TE1 cell lines. In fact, PPP2R2A refers to a large family of heterotrimeric Ser/Thr phosphatases and can be considered a tumor suppressor gene that regulates tumor growth. Studies have found that PPP2R2A suppresses gastric cancer cell proliferation, invasion, and epithelial-mesenchymal transition (EMT)[35]. TNP1, also known as transition protein 1, has been confirmed to be expressed in murine Leydig tumor cell lines[36]. In addition, a previous study reported that overexpression of ZNF350 in colon cancer cells significantly increased their proliferative and migratory abilities[37]. In summary, these results suggest that the DDRG signature might be exploited as a viable ESCC prognostic indicator. Additionally, we also explored the critical signature of DDRGs in hopes of providing evidence for immunotherapy. A previous study reported that DDR can reshape the tumor microenvironment[38]. In this study, we found that the infiltration levels of naive CD4 T cells, activated mast cells and monocytes were significantly higher in the low-risk group. Naive CD4 T cells are a type of lymphocyte, and previous studies have investigated their prognostic value in tumors[39, 40]. Yang et al.[41] and Hara et al.[42] reported that increased naive CD4 T cells can predict favorable survival in resectable NSCLC. Mast cells produce a unique set of antitumorigenic immune mediators, which has been proven in a tumor model[43]. Plotkin et al. revealed that human mast cells can release large amounts of GM-CSF[44]. GM-CSF has been demonstrated to suppress tumor cell proliferation and has been applied in clinical antitumor therapies[45]. Additionally, Fereydouni et al. presented evidence that mast cells can be polarized to regulate their hyperinflammatory and antitumor effects as potential target cells for precision immunotherapy[46]. Interestingly, activated NK cells were lower in the low-risk group. Activated NK cells are a phenotype of NK cells, and studies have demonstrated that a lower proportion of activated NK cells promotes the level of tumor infiltration to favor the formation of the immune microenvironment[47]. Immunosuppressive gene analysis revealed that the DDRG signature was positively correlated with BTLA, CTLA4, HAVCR2, IL10RB, PDCD1LG2, and TIGIT, particularly PDCD1LG2, which may provide clues for acting as a potential immune target to enhance antitumor effects in ESCC.

This study has several limitations. First, consensus clustering, the development of a prognostic model, and validation were performed based on data sources. The potential for selection bias is inevitable. Independent validation data from multiple centers and a larger sample size are required to validate these findings. Second, the number of DDRGs was limited, and additional DDRGs identified in new studies have not been included in the present study. Third, the correlation between the DDRG signature and immunotherapy response in ESCC was not evaluated directly because public database information from ESCC patients receiving immunotherapy was not available. Further studies will be performed to collect clinical and experimental data and investigate the mechanisms of DDR molecular subtypes and the DDR prognostic model on the prognosis of ESCC patients receiving immunotherapy.

## Conclusion

In summary, our study comprehensively explored the predictive efficacy of the DDRG signature on the survival of ESCC patients. Additionally, immune-related analysis revealed that the DDRG signature could distinguish immune activity to screen appropriate patients for benefitting from immunotherapy. This is the first report of prognostic and immune analysis based on the DDR gene signature for ESCC, which might facilitate individual management in the era of accurate immunotherapy.

## Supplementary Information

**Additional file 1**. ** Figure S1:** Establishment of prognostic model in ESCC. (A) LASSO coefficients of the five prognostic DDRGs. (B) Identifying LASSO deviance profiles using cross-validation. (C) The forest plot shows prognostic DDRGs using univariate Cox regression analysis. (D) The five prognostic DDRGs and 50 related genes. ESCC, esophageal squamous cell carcinoma; DDRGs, DNA damage repair genes.

**Additional file 2**. ** Figure S2**: GSEA results of specific enrichment set. (A, B) The 10 significantly enriched GO terms in high and low-risk ESCC patients. (C, D) KEGG pathways of different risk groups. GSEA, gene set enrichment analysis; GO, Gene Ontology; KEGG, Kyoto Encyclopedia of Genes and Genomes; ESCC, esophageal squamous cell carcinoma

## Data Availability

The datasets analyzed during the current study are available in the GEO database (https://www.ncbi.nlm.nih.gov/geo/) (PERSISTENT ACCESSION NUMBER TO DATASETS: GSE53625) and TCGA database (https://portal.gdc.cancer.gov/) (PERSISTENT ACCESSION NUMBER TO DATASET: TCGA-ESCC). All data generated or analyzed during this study are included in this published article and its supplementary information files.
