# Supplementary material for: A novel DNA damage repair gene-related prognostic model for evaluating the prognosis and tumor microenvironment infiltration of esophageal squamous cell carcinoma
Source: BMC Med Genomics. 2023 Feb 20;16:27. doi: 10.1186/s12920-023-01459-1 (PMC9940400; doi:10.1186/s12920-023-01459-1)

**Additional file 1: Fig. 1**. Establishment of prognostic model in ESCC. (A) LASSO coefficients of the five prognostic DDRGs. (B) Identifying LASSO deviance profiles using cross-validation. (C) The forest plot shows prognostic DDRGs using univariate Cox regression analysis. (D) The five prognostic DDRGs and 50 related genes. ESCC, esophageal squamous cell carcinoma; DDRGs, DNA damage repair genes.


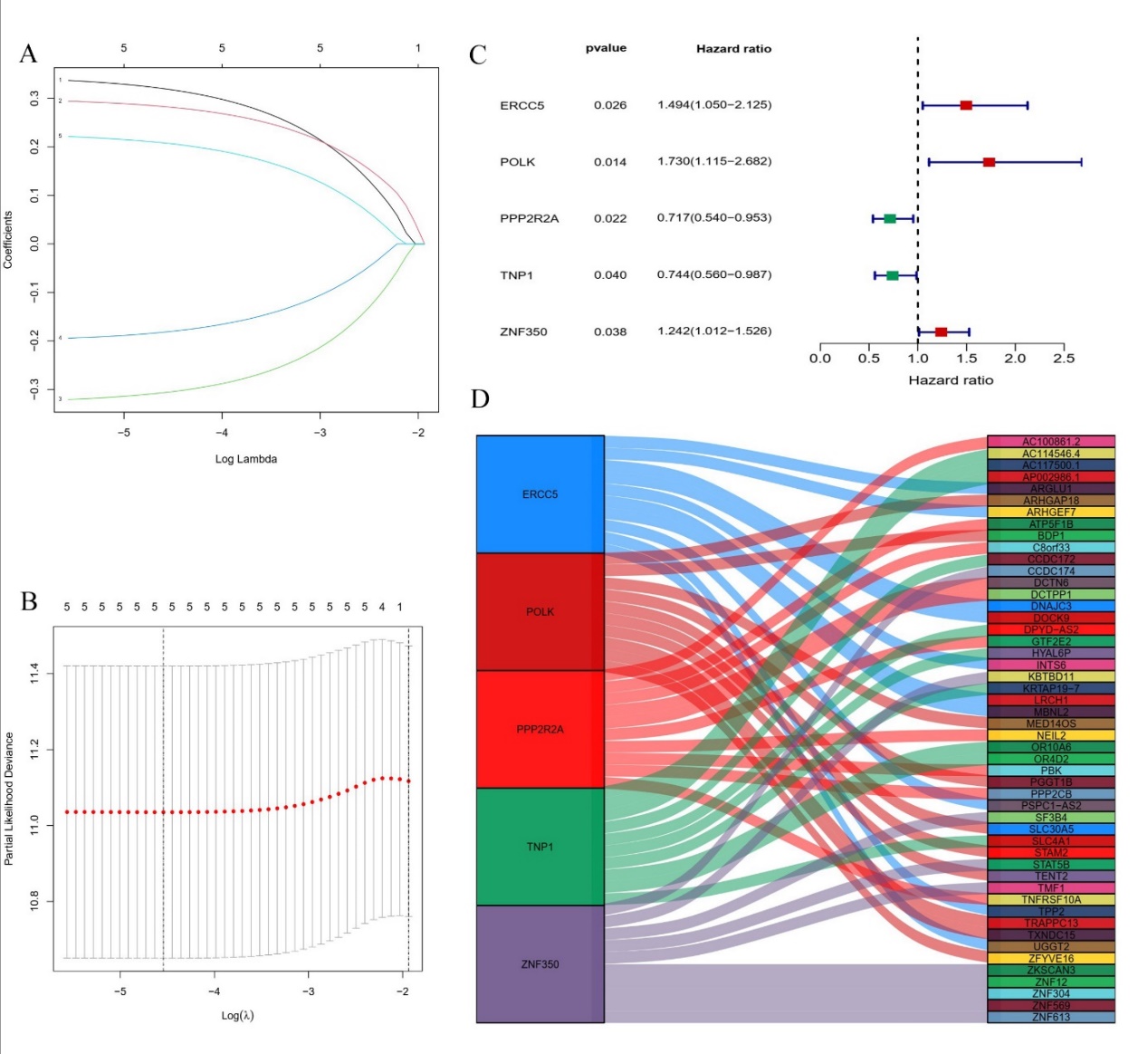

Supplement: Supplementary file 1 — Additional file 1. Figure S1: Establishment of prognostic model in ESCC. (A) LASSO coefficients of the five prognostic DDRGs. (B) Identifying LASSO deviance profiles using cross-validation. (C) The forest plot shows prognostic DDRGs using univariate Cox regression analysis. (D) The five prognostic DDRGs and 50 related genes. ESCC, esophageal squamous cell carcinoma; DDRGs, DNA damage repair genes. [file 12920_2023_1459_MOESM1_ESM.docx]
