# Supplementary material for: A novel DNA damage repair gene-related prognostic model for evaluating the prognosis and tumor microenvironment infiltration of esophageal squamous cell carcinoma
Source: BMC Med Genomics. 2023 Feb 20;16:27. doi: 10.1186/s12920-023-01459-1 (PMC9940400; doi:10.1186/s12920-023-01459-1)

**Additional file 2: Fig. 2**. GSEA results of specific enrichment set. (A, B) The 10 significantly enriched GO terms in high and low-risk ESCC patients. (C, D) KEGG pathways of different risk groups. GSEA, gene set enrichment analysis; GO, Gene Ontology; KEGG, Kyoto Encyclopedia of Genes and Genomes; ESCC, esophageal squamous cell carcinoma.


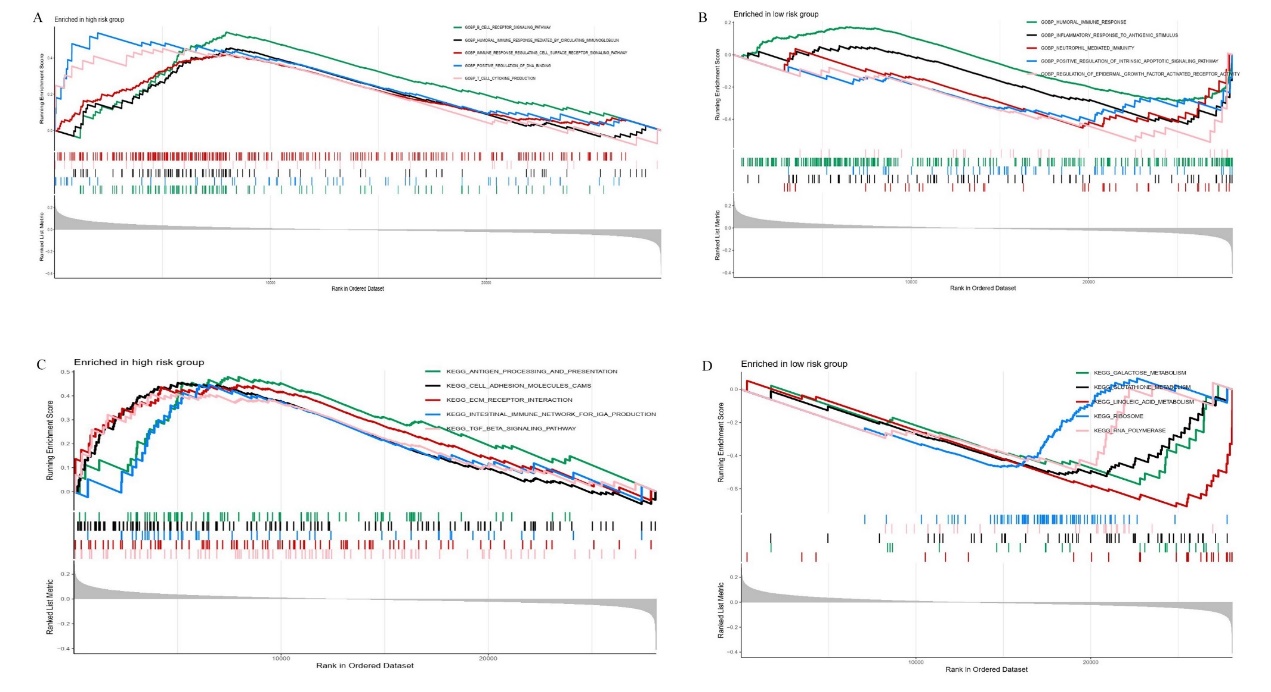

Supplement: Supplementary file 2 — Additional file 2. Figure S2: GSEA results of specific enrichment set. (A, B) The 10 significantly enriched GO terms in high and low-risk ESCC patients. (C, D) KEGG pathways of different risk groups. GSEA, gene set enrichment analysis; GO, Gene Ontology; KEGG, Kyoto Encyclopedia of Genes and Genomes; ESCC, esophageal squamous cell carcinoma [file 12920_2023_1459_MOESM2_ESM.docx]
